# Intracellular Accumulation as an Indicator of Cytotoxicity to Screen Hepatotoxic Components of *Chelidonium majus* L. by LC–MS/MS

**DOI:** 10.3390/molecules24132410

**Published:** 2019-06-29

**Authors:** Cuiting Wu, Xin Wang, Ming Xu, Youping Liu, Xin Di

**Affiliations:** 1 Laboratory of Drug Metabolism and Pharmacokinetics, Shenyang Pharmaceutical University, 103 Wenhua Road, Shenyang 110016, China; 2 Shenyang Analytical Application Center, Shimadzu (China) Co. Ltd., 167 Qingnian Street, Shenyang 110016, China

**Keywords:** intracellular accumulation, LC–MS/MS, alkaloids, hepatotoxicity, *Chelidonium majus* L., cytotoxicity

## Abstract

A novel strategy was developed to identify hepatotoxic compounds in traditional Chinese medicines (TCMs). It is based on the exposure of HL-7702 cells to a TCM extract, followed by the identification and further determination of potential hepatotoxic compounds accumulated in the cells by liquid chromatography–tandem mass spectrometry (LC–MS/MS). As a case study, potential hepatotoxic components in *Chelidonium majus* L. were screened out. Five alkaloids (sanguinarine, coptisine, chelerythrine, protopine, and chelidonine) were identified by LC–MS/MS within 10 min, and their intracellular concentrations were first simultaneously measured by LC–MS/MS with a run time of 4 min. A cell viability assay was performed to assess the cytotoxicity of each alkaloid. With their higher intracellular concentrations, sanguinarine, coptisine, and chelerythrine were identified as the main hepatotoxic constituents in *Ch. majus*. The study provides a powerful tool for the fast prediction of cytotoxic components in complex natural mixtures on a high-throughput basis.

## 1. Introduction

Traditional Chinese medicines (TCMs) have long played an important role in health care. The therapeutic efficacy of TCMs has been proven by medical practices in China and has also been confirmed in many countries worldwide. However, the frequent adverse reactions of TCMs have raised public concern on the safety and toxicity of TCMs [1]. Although the adverse reactions caused by TCMs are mostly transient and reversible with the cessation of treatment, their hepatic adverse effects may be severe and sometimes even permanent [2]. In recent years, liver injury cases associated with the use of TCMs have increasingly been reported [3]. Examples of some known hepatotoxic TCMs are *Polygonum multiflorum*, *Dioscorea bulbifera*, *Dichroa febrifuga*, *Mistletoe Viscum*, *Melia azedarach*, *Tripterygium wilfordii*, *Gynura segetum,* and *Senecio vulgaris*. At present, the mechanisms of liver injury induced by TCMs are still not very clear. Fortunately, some specific phytochemical ingredients that may cause liver injury have been identified, such as diterpenoid-lactones in *T. wilfordii* and *D. bulbifera* and pyrrolizidine alkaloids in *G. segetum* and *S. vulgaris* [1]. The identification of hepatotoxic components in TCMs would be helpful to elucidate the hepatoxicity basis of TCMs and the mechanisms of TCMs-induced liver injury.

The traditional strategy to screen toxic components from TCMs often involves the isolation and purification of TCM components and their subsequent in vitro or in vivo toxicity evaluation [4]. These experimental approaches are very time- and resource-consuming [5]. With the advantages of time-efficiency and cost-effectiveness, in silico screening methods based on quantitative structure–toxicity relationship (QSAR) analysis have been used as a complementary tool to identify hepatotoxic compounds in TCMs [6,7]. Nevertheless, a common drawback of the above experimental and computational methods is that the hepatotoxicity of each compound is evaluated individually with ignorance of the synergistic effects of multiple constituents in TCMs. Recently, fingerprint-toxicity modeling methods have been employed to screen the major hepatotoxic components in *Euodia rutaecarpa*, *D. bulbifera*, and *P. multiflorum* [8,9,10]. These methods are based on the characterization of the correlations between characteristic chromatographic peaks and the holistic hepatotoxicity of TCMs by multivariate statistical analysis. However, these methods suffer from the disadvantages of extensive workload and high cost (for example, linked to the increased use of animals).

In recent years, screening methods based on live cell extraction and high-performance liquid chromatography–mass spectrometry analysis have been applied to identify potential bioactive components in TCMs [11,12,13]. These methods demonstrated the advantages of high efficiency and specificity over previous methods. Inspired by these methods, hepatotoxic components in TCMs could also be screened using cell-based screening strategies. Unlike the screening of bioactive components of TCMs, however, the screening of hepatotoxic components should not be restricted to the identification of the compounds accumulated in hepatocytes. A further determination of intracellular accumulation of the identified compounds is necessary. It is well known that the dose makes the poison. Drug-induced toxicity is thought to be mediated by the drug accumulation in target cells. Intracellular drug concentrations were reported to have significant impact on cytotoxicity [14]. It remains to be seen whether intracellular accumulation can be used as an indicator of cytotoxicity for a fast screening of hepatotoxic components in TCMs.

In the present study, we attempted to develop a whole-cell-based strategy to screen and predict potential hepatotoxic components in *Chelidonium majus* L., a perennial herbaceous plant of the family *Papaveraceae. Ch. majus*, also known as greater celandine, is widely used in Asia and Europe. It has been used as a folk medicine due to its various pharmacological effects [15], such as antibacterial, anti-inflammatory, and anti-tumour actions [16,17]. Isoquinoline alkaloids are the main chemical components of *Ch. majus*, among which sanguinarine, coptisine, and chelerythrine showed cytotoxicity in rat hepatocytes [18]. *Ch. majus* may also induce liver injury after long-term administration in large doses [19]. To identify the components contributed to the holistic hepatotoxicity of *Ch. majus*, the proposed strategy involved: (1) exposing HL-7702 cells to *Ch. majus* extract; (2) identifying the compounds accumulated in HL-7702 cells; (3) determining the intracellular accumulation of potential hepatotoxic compounds. Liquid chromatography–tandem mass spectrometry (LC–MS/MS) was used for the identification of hepatotoxic components and the determination of their intracellular accumulation. 

## 2. Results and Discussion

### 2.1. Selection of Hepatic Cell Line

Human primary hepatocytes are the ideal cell model for the prediction of hepatotoxicity in vitro. However, their very limited proliferative capacity restricts their use in high-throughput screenings. HepG2, an immortalized cell line consisting of human liver carcinoma cells, is suitable for a high-throughput screening strategy [20]. However, HepG2 cells do not reflect the normal liver’s physiological response to toxic substances. HL-7702 is an immortalized cell line derived from normal human hepatocytes retaining many features of primary hepatocytes and, therefore, was used for the prediction of hepatotoxicity in the present study.

### 2.2. Optimization of Cell Lysate Preparation

In order to accurately identify the components accumulated in HL-7702 cells, interferences from extracellular compounds should be eliminated. An appropriate method of cell washing could eliminate the interferences. In preliminary experiments, cycles of cell washing (3, 6, and 9) after incubation were investigated using PBS(-) as the washing solution. Each eluate was collected for LC–MS/MS analysis. No substances from *Ch. majus* were detected in the ninth eluate, indicating that nine washes removed the unbound compounds from the cell surface. It was speculated that some of the compounds may remain in cell–cell junctions. To prove this, the cells were first washed three times with PBS(-) to rinse off the medium containing *Ch. majus*. Then, trypsin was used to break the cell–cell junctions. The cell suspension was centrifuged, and the cell pellets were washed with PBS(-) for additional three times. Surprisingly, no substances from *Ch. majus* were detected in the last eluate. In this way, cycles for cell washing were reduced by a third.

Dilution of the cell lysate before quantitative analysis by LC–MS/MS was required to reduce matrix effects and thus to improve the reproducibility and accuracy of the method. Different dilution ratios with acetonitrile were investigated, and a dilution ratio of 7:1 was found to be sufficient to eliminate the matrix effects while maintaining acceptable sensitivity.

### 2.3. Optimization of LC–MS/MS Conditions

In order to achieve a fast determination of the intracellular accumulation of *Ch. majus* compounds, gradient mobile phase chromatography was optimized using an ACQUITY UPLC C18 column (2.1 × 50 mm, 1.7 µm) instead of the common Diamonsil C18 column (150 × 4.6 mm I.D., 5 µm) that we previously used for simultaneous LC−MS/MS determination of five isoquinoline alkaloids in rat plasma [21]. As cross-talk effect between the multiple reaction monitoring (MRM) transitions of protopine and chelidonine was observed, and baseline separation of the isomers was required. In comparison with the methanol/water mobile phase, the acetonitrile/water mobile phase provided a better resolution of the isomers in a shorter run time. Addition of 0.1% formic acid to the acetonitrile/water mobile phase could improve peak shapes and increase MS detection sensitivity. Finally, 20% acetonitrile in water (containing 0.1% formic acid) was used as the initial mobile phase, maintained for 1 min, then increased up to 40% acetonitrile in 2.5 min, and finally returned to 20% acetonitrile in 0.5 min. The total chromatographic run time was only 4 min.

### 2.4. Method Validation

Validation of the method was carried out according to FDA Bioanalytical Method Validation, Guidance for Industry [22]. 

#### 2.4.1. Specificity and Crosstalk Effect

**Figure 1:**
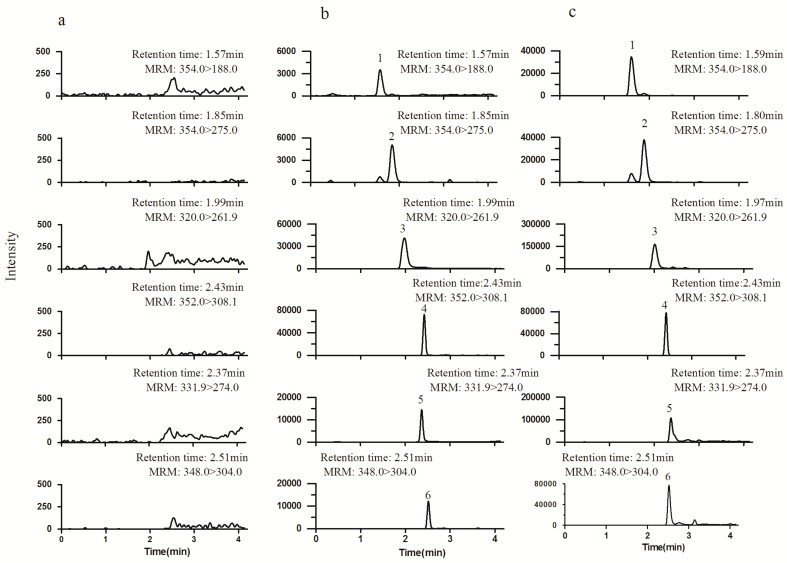
Representative multiple reaction monitoring (MRM) chromatograms of blank cell lysates (**a**), blank cell lysates spiked with standard solutions (**b**), and cell lysates collected after coincubation with *Chelidonium majus* for 10 min (**c**). Peak 1: protopine; 2: chelidonine; 3: coptisine; 4: palmatine (internal standard, IS); 5 sanguinarine; 6: chelerythrine.

The specificity of the method was investigated by analyzing blank cell lysates to check matrix interference. Crosstalk between the incompletely resolved compounds was evaluated by monitoring the MRM transitions of the compounds following individual injections of a high-concentration solutions of the analytes and internal standard (IS). Representative chromatograms are shown in Figure 1. No peaks from the blank lysates were observed at the retention time of the analytes and IS, and no cross-talk effect was observed.

#### 2.4.2. Linearity, LLOQ, and ULOQ

The linearity of the method was analyzed in duplicate at each concentration level over three consecutive days. The calibration curve was obtained by plotting the ratio between the peak area of each analyte and that of IS (*y*) versus the nominal concentration (*x*). Each calibration curve was fitted by least-squares linear regression using 1/*x^2^* as a weighing factor. The lower limit of quantification (LLOQ) was defined as the lowest concentration and tested by six replicate analyses. The results of linear regression, LLOQ, and upper limit of quantification (ULOQ) are listed in Table 1. All calibration curves presented good linearity, with correlation coefficients (*r*) greater than 0.9949. The LLOQs and ULOQs were determined with acceptable precision and accuracy, presenting a relative standard deviation (RSD) lower than 4.9% and 8.7% and a relative error (RE) in the range of −8.2% to 6.8% and −4.6% to 6.7%, respectively.

#### 2.4.3. Precision and Accuracy

The precision and accuracy of the method were assessed by analyzing LLOQ and quality control (QC) samples with six replicates at three QC levels on three validation days. The accuracy was calculated as percent deviation of the measured concentration from the nominal concentration (RE). Intra- and inter-day precisions were evaluated using one-way analysis of variance (ANOVA). The results of precision and accuracy are shown in Table 2. The precisions ranged from 1.3% to 7.5%, and the accuracy was between −10.5% and 5.5%.

#### 2.4.4. Extraction Recovery and Matrix Effect

Extraction recovery was evaluated at three QC concentration levels using six replicates at each concentration by comparing the peak areas of the analytes and IS obtained from the QC samples with those of post-extraction spiked samples. The matrix effect was evaluated at three QC levels by comparing the peak areas of the analytes spiked after extraction with those of the pure standard solutions at the same concentration. The results of extraction recovery and the matrix effect are shown in Table 3. The extraction recovery was more than 80% for all the analytes and about 90% for IS. No significant matrix effect was observed for all compounds, with the values at three QC levels in the range of 87.5–107.2%.

#### 2.4.5. Stability

The stability of the analytes was evaluated at low and high concentration levels under a variety of storage and process conditions, including post-preparative stability (24 h at autosampler, 4 °C), bench-top stability (12 h at room temperature), long-term stability (−80 °C for 30 days), and freeze–thaw stability (three freeze–thaw cycles). The results of stability are shown in Table 4. The analytes were stable under the tested storage and process conditions.

### 2.5. Identification of Ch. majus Components

**Figure 2:**
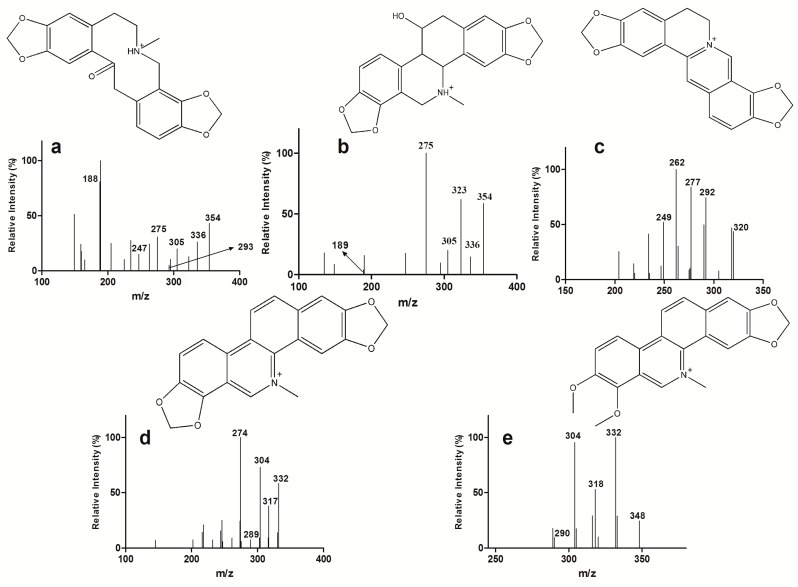
Full-scan product ion mass spectra of the [M + H]^+^ ions of protopine (**a**) and chelidonine (**b**) and the [M]^+^ ions of coptisine (**c**), sanguinarine (**d**), chelerythrine (**e**) in cell lysates after coincubation with *Ch. majus* for 30 min.

**Figure 3:**
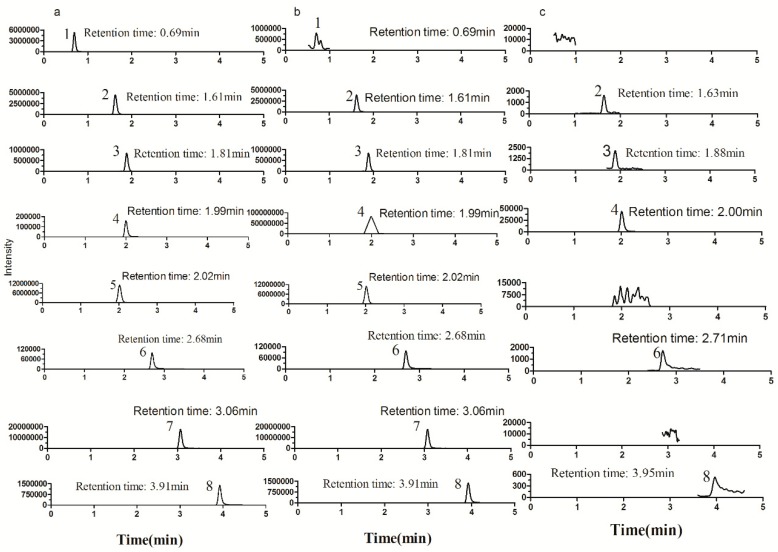
Typical full-scan product ion chromatograms of standard solutions (**a**), extract from crude samples (**b**), and cell lysates after incubation with *Ch. majus* for 30 min (**c**). Peak 1: magnoflorine; 2: protopine; 3: chelidonine; 4 coptisine; 5: allocryptopine; 6: sanguinarine; 7: berberine; 8: chelerythrine.

The main components in the extract of *Ch. majus* were first identified by LC–MS/MS. Eight alkaloids (magnoflorine, protopine, chelidonine, coptisine, allocryptopine, sanguinarine, berberine, chelerythrine) were confirmed by comparing their retention times and MS/MS fragmentation pathways with those obtained from reference standards. After exposing HL-7702 cells to the extract of *Ch. majus* (2.5 mg/mL of raw herb) for 30 min, five of the eight alkaloids (protopine, chelidonine, coptisine, sanguinarine, and chelerythrine) were detected in the cell lysate. The retention times and MS^2^ data of eight alkaloids are listed in Table 5. The full-scan product ion chromatograms of the reference standards, *Ch. majus* extract, and cell lysate are shown in Figure 2 and Figure 3.

### 2.6. Determination of Intracellular Accumulation

**Figure 4:**
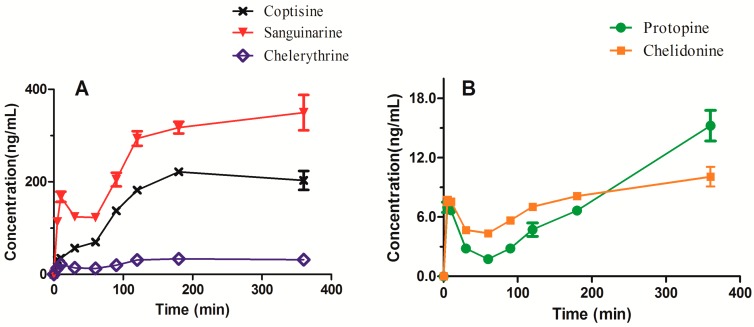
Intracellular concentrations of (**A**) coptisine, sanguinarine and chelerythrine, and (**B**) protopine and chelidonine after coincubation with *Ch. majus* extract.

The intracellular concentrations of the five alkaloids, after exposing HL-7702 cells to the extract of *Ch. majus* (2.5 mg/mL of raw herb) for 0–360 min, were determined by LC–MS/MS. Figure 4 shows the intracellular concentration–time curves of the five alkaloids. It can be seen that the intracellular concentrations of the alkaloids increased with the increase of the exposure time and reached a plateau at 180 min. The highest intracellular accumulation was observed for sanguinarine, followed by coptisine and chelerythrine. These three alkaloids are quaternary benzo[*c*]phenanthridine alkaloids, and their intracellular concentrations were far higher than those of protopine and chelidonine at equilibrium. For example, the intracellular concentration of sanguinarine was approximately 30-fold higher than that of chelidonine. Surprisingly, the content of sanguinarine in the extract of *Ch. majus* was slightly lower than that of chelidonine (3.30 versus 4.04 mg/g of dry extract). Chelerythrine showed the third highest intracellular accumulation, despite its content in the extract being the lowest of the five alkaloids (only 0.12 mg/g of dry extract). The results revealed that there was no correlation between the intracellular accumulation of the alkaloids and the contents of the alkaloids in the extract of *Ch. majus*. High accumulation of a substance in cells is mainly due to high permeability and/or high affinity for influx transporters.

Our results of the intracellular accumulation of sanguinarine, coptisine, and chelerythrineare are consistent with the hepatotoxicity data reported previously [18,23,24]. Also, sanguinarine produced greater toxicity than chelerythrine [25]. Therefore, we speculated that these three alkaloids were the main hepatotoxic constituents of *Ch. majus*. The hepatotoxicity of *Ch. majus* should be mediated mainly by their accumulation in hepatocytes.

### 2.7. Cell Viability Assay

**Figure 5:**
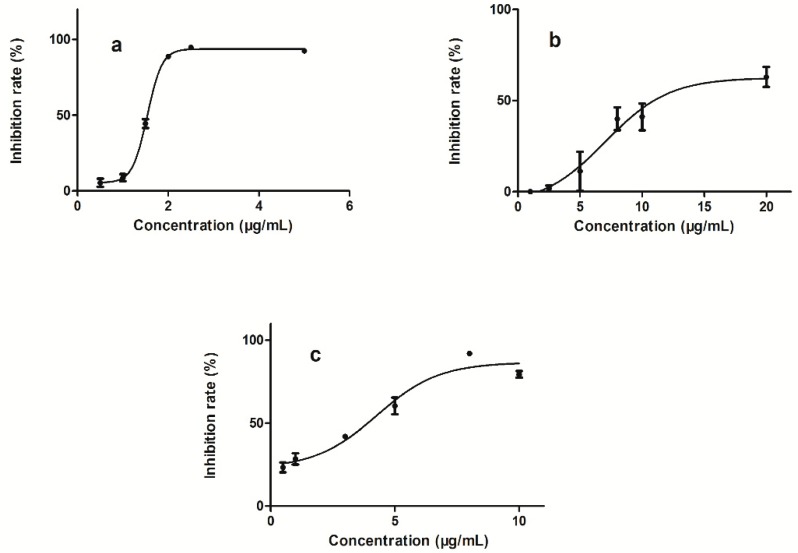
Cell viability inhibitory effects in HL-7702 cells treated with sanguinarine (**a**), coptisine (**b**), chelerythrine (**c**).

The effects of five alkaloids on the cell viability of HL-7702 cells were individually investigated by CCK-8 assay. Figure 5 shows the inhibitory effects of sanguinarine, coptisine, and chelerythrine on cell viability. The IC_50_ values for sanguinarine, chelerythrine, and coptisine were 1.54 ± 0.05, 4.54 ± 0.66, and 8.19 ± 3.28 µg/mL, respectively. IC_50_ was not reached up to the concentration of 20 µg/mL for protopine and chelidonine. The IC_50_ of sanguinarine was the lowest, whereas the intracellular accumulation of sanguinarine was the highest. Chelerythrine had lower IC_50_ than coptisine, yet it did not show higher intracellular accumulation than coptisine. This can be explained by the possibility that cell viability was evaluated individually for each alkaloid, with ignorance of the synergistic effects of multiple constituents in TCMs. Intracellular accumulation could reflect the holistic hepatotoxicity of *Ch. majus* and therefore could be used as a potential indicatorof cytotoxicity to screen hepatotoxic components of *Ch. majus*.

## 3. Materials and Methods 

### 3.1. Chemicals and Materials

*Ch. majus* was obtained from Tongrentang Chinese Medicne (Shenyang, China). The reference standards of protopine, allocryptopine, chelerythrine, chelidonine, and magnoflorine were purchased from Shenzhen Medherb Biotechnology Co., Ltd. (Shenzhen, China). Sanguinarine and coptisine were obtained from Chengdu Mansite Pharmaceutical Co., Ltd. (Sichuan, China). The IS, palmatine, was purchased from the National Institute for the Control of Pharmaceutical and Biological Products (Beijing, China). Trypsin and RPMI-1640 medium were purchased from Gibco Life Technologies Corporation (Grand Island, NY, USA), and newborn calf serum from Gibco Life Technologies Corporation (Auckland, New Zealand). Penicillin, streptomycin, and PBS(-) were obtained from Sangon Biotech (Shanghai, China). Dimethyl sulphoxide (DMSO) was obtained from Tianjin Bodi Chemical Co., Ltd. (Tianjin, China). The commercial Cell Counting Kit-8 (CCK8) was obtained from Dojindo (Kumamoto, Japan). All of the cell culture plastics were bought from Corning incorporated (Corning, NY, USA). HL-7702 cells were bought from the Cell Bank of the Chinese Academy of Sciences (Shanghai, China). Acetonitrile and formic acid of HPLC grade were obtained from Sigma-Aldrich (St. Louis, MO, USA) and Dikma Technologies (Lake Forest, CA, USA), respectively. Water used throughout the study was prepared by a Millipore Milli-Q Academic system (Millipore, Bedford, MA, USA). All other reagents were of analytical grade.

### 3.2. Preparation of Calibration Standards and QC Samples

Stock solutions of each analyte were prepared in acetonitrile/water (1:1, *v*/*v*). A mixed stock solution was prepared by combining appropriate aliquots of each stock solution and diluting with acetonitrile/water (1:1, *v*/*v*). A series of mixed working solutions were made by successively diluting the mixed stock solution with acetonitrile/water. An IS solution was prepared at the concentration of 80 ng/mL. QC solutions were made from a separately prepared mixed stock solution. All solutions were stored at 4 °C before use.

Calibration standards were prepared daily by spiking each mixed working solution into blank cell lysates to yield the desired final concentrations. QC samples were prepared by the same procedures to yield low, medium, and high final concentrations.

### 3.3. Sample Preparation

The powder of *Ch. majus* was extracted with five-fold (*w*/*v*) 70% ethanol for 1 h three times in an ultrasonic bath and then evaporated to dryness. The dried extract was dissolved in DMSO and filtered through a 0.22 μm cellulose acetate membrane. The filtrate was diluted with RPMI-1640 medium to yield a final concentration equivalent to 2.5 mg/mL of raw herb.

### 3.4. Cell Culture

HL-7702 cells were dissociated with 0.05% trypsin–EDTA and subcultured in 10 cm-diameter dishes with RPMI-1640 medium containing 10% newborn calf serum, 100 µg/mL streptomycin, and 100 U/mL penicillin. The cells were maintained at 37 °C in a humidified atmosphere of 5% CO_2_ and 95% air. The culture medium was replaced every 48 h and removed until the cells reached about 80% confluence.

### 3.5. Cell Exposure to the Test Sample

HL-7702 were incubated in the medium containing *Ch.majus* for 30 min. Then, the cells were washed and lysed (following the procedures described below). The cell lysate was used for the identification study. HL-7702 were incubated in blank medium and in medium containing *Ch. majus* for 5, 10, 30, 60, 90, 120, 180, and 360 min. Then, the cells were washed and lysed for the determination study.

### 3.6. Cell Lysate Preparation

After incubation, the cells were washed three times with PBS(-) and then harvested with 0.05% trypsin–EDTA. The cell suspension (about 5 × 10^6^ cells) was centrifuged at 1000 rpm for 5 min and further washed three times with PBS(-). The cell pellets were lysed in 200 µL of ice-cold methanol by vortex-mixing for 2 min. The cell lysate was centrifuged at 12,000 rpm for 4 min. To identify the compounds accumulated in the cells, 2 µL of supernatant was directly injected into the LC–MS/MS system. For the purpose of determining the intracellular accumulation of the compounds, 20 µL of supernatant was placed into a 1.5 mL Eppendorf tube and then 20 µL of IS working solution and 100 µL of acetonitrile were added. The mixture was vortex-mixed for 1 min. A 2 µL aliquot of the mixture was injected into the LC–MS/MS system.

### 3.7. LC–MS/MS Analysis

LC–MS/MS analysis was performed on a Shimadzu Nexera LC system coupled with a Shimadzu LCMS-8050 triple quadrupole tandem mass spectrometer equipped with an ESI interface in positive ionization mode (Shimadzu, Kyoto, Japan). Chromatographic separation was performed on an ACQUITY UPLC C18 column (2.1 × 50 mm, 1.7 µm) maintained at 40 °C by gradient elution, using water containing 0.1% formic acid (solvent A) and acetonitrile containing 0.1% formic acid (solvent B) as the mobile phase at a flow rate of 0.3 mL/min. The gradient program for compound identification was: 0–4.0 min, 20%–30% B, 4.0–8.0 min, 30%–90% B, 8.0–10.0 min, 20% B. The gradient program for compound quantitation was: 0–1.0 min 20% B, 1.0–3.5 min, 60% B, 3.5–4.0 min, 20% B. The MS conditions were set as follows: nebulizing gas flow, 3.0 L/min; drying gas flow, 10.0 L/min; heat-block temperature, 400 °C; desolvation line temperature, 250 °C; interface voltage, 4 kV. Product ion scan and MRM mode were used for identification and quantitation, respectively. The ion transitions and collision energies are listed in Table 6. Data acquisition and processing were conducted using LabSolutions LCMS Ver.5.82 SP1 (Shimadzu, Kyoto, Japan).

### 3.8. Cytotoxicity Assay

Cell viability was performed using the Cell Counting Kit-8 (CCK-8) assay according to the manufacturer’s instructions. In brief, HL-7702 cells were seeded in a 96-well plate at a density of 25,000 per well. After 24 h, the cell growing medium was removed and replaced with fresh medium containing *Ch. majus* or the test compound at various concentrations. After an additional 24 h, 10 µL of WST reagent was added into each well and incubated with the cells for 2 h. The optical density of each well was measured by a microplate reader (Corona Electric, Ibaraki-Ken, Japan) at a wave length of 450 nm.

## 4. Conclusions

A novel strategy based on the use of intracellular accumulation as an indicator of cytotoxicity to screen hepatotoxic components in TCMs has been developed. The simultaneous quantification of multiple components of TCMs in cells was achieved for the first time by LC–MS/MS. This fast and sensitive method was successfully applied to determine five potential hepatotoxic alkaloids accumulated in HL-7702 cells. Sanguinarine, coptisine, and chelerythrine were identified as the main hepatotoxic constituents of *Ch. majus* on the basis of their intracellular accumulation and IC_50_ values. Our developed strategy has the advantages of high efficiency and low cost and is especially informative, since it takes into consideration the synergistic effects of multiple constituents of TCMs. Further investigation of the synergistic effects will be carried out by comparing the intracellular accumulation of herbal extracts with those of individual standards. This method provides a powerful tool to screen potential hepatotoxic components of TCMs, obtaining comprehensive data about their accumulation in cells, which could be a potential predictor of cytotoxicity.

## Figures and Tables

**Table 1:** Regression equation, linear range, lower limit of quantification (LLOQ), and upper limit of quantification (ULOQ) of five alkaloids in cell lysates by LC–MS/MS analysis. RE: relative error.

Compound	Regression Equation	*r*	Linear Range (ng/mL)	LLOQ		ULOQ	
				Found Concentration (ng/mL)	RE (%)	Found Concentration (ng/mL)	RE (%)
Protopine	*y* = 1.6558*x* + 0.0078	0.9992	0.75–30	0.728	−2.9	28.62	−4.6
Chelidonine	*y* = 4.2789*x* + 0.0087	0.9987	0.5–20	0.495	−1.0	20.48	2.4
Coptisine	*y* = 1.1156*x* + 0.0399	0.9992	12.5–500	13.35	6.8	533.5	6.7
Sanguinarine	*y* = 0.1732*x* + 0.0784	0.9989	20.0–800	20.72	3.6	770.4	−3.7
Chelerythrine	*y* = 0.2818*x* − 0.0068	0.9949	6.0–240	5.508	−8.2	252.7	5.3

**Table 2:** Precision and accuracy of the determination of five alkaloids in cell lysates by LC–MS/MS analysis. RSD: relative standard deviation.

Compound	Nominal Concentration (ng/mL)	Intra-Day			Inter-Day		
		Found Concentration (ng/mL)	RE (%)	RSD (%)	Found Concentration (ng/mL)	RE (%)	RSD (%)
Protopine	0.75	0.776	3.5	4.7	0.772	2.9	6.5
	1.875	1.978	5.5	3.4	1.954	4.2	4.1
	6.0	5.91	−1.5	4.2	5.934	−1.1	2.4
	22.5	21.02	−6.6	3.0	21.22	−5.7	3.8
Chelidonine	0.5	0.526	5.2	3.7	0.523	4.6	7.1
	1.25	1.295	3.6	4.1	1.319	5.5	2.2
	4.0	3.860	−3.5	2.9	3.916	−2.1	3.5
	15	13.74	−8.4	3.0	13.85	−7.7	2.8
Coptisine	12.5	12.74	1.9	4.8	12.96	3.7	5.9
	31.25	32.75	4.8	5.0	31.97	2.3	7.5
	100	97.9	−2.1	3.4	99.70	−0.3	4.1
	375	363.4	−3.1	3.7	367.1	−2.1	2.1
Sanguinarine	20	20.54	2.7	3.8	20.72	3.6	4.2
	50	52.45	4.9	2.6	51.85	3.7	3.9
	160	155.2	−3.0	4.3	156.2	−2.4	1.5
	600	567.6	−5.4	3.7	583.8	−2.7	6.0
Chelerythrine	6.0	5.604	−6.6	7.3	5.766	−3.9	6.7
	15	13.54	−9.7	3.2	13.43	−10.5	3.9
	48	43.78	−8.8	2.8	44.06	−8.2	1.3
	180	187.6	4.2	3.8	181.6	0.9	4.6

**Table 3:** Extraction recovery and matrix effect of five alkaloids and IS in cell lysates for LC–MS/MS analysis.

Compound	Nominal Concentration	Extraction Recovery		Matrix Effect	
	(ng/mL)	Mean (%)	RSD (%)	Mean (%)	RSD (%)
Protopine	1.875	108.7	13.4	93.5	12.1
	6.0	90.2	8.0	99.9	3.1
	22.5	96.3	10.1	107.2	3.0
Chelidonine	1.25	98.3	7.7	89.4	1.6
	4.0	90.8	9.4	101.7	4.4
	15.0	101.1	9.4	106.7	2.6
Coptisine	31.25	88.7	5.2	91.8	2.1
	100	81.3	8.3	96.4	10.3
	375	96.1	8.5	107.1	7.0
Sanguinarine	50	88.2	10.1	87.5	1.5
	160	90.0	9.1	98.1	2.8
	600	99.6	7.4	105.9	2.7
Chelerythrine	15	98.6	12.1	90.7	1.8
	48	92.9	3.4	104.2	2.9
	180	92.1	10.9	106.7	3.3
Palmatine (IS)	32	92.6	8.9	100.5	5.5

**Table 4:** Stability of five alkaloids under the tested conditions.

Compound	Nominal Concentration (ng/mL)	Freeze–Thaw Stability		Long-Term Stability		Bench-Top Stability		Post-Preparative Stability	
		RE (%)	RSD (%)	RE (%)	RSD (%)	RE (%)	RSD (%)	RE (%)	RSD (%)
Protopine	1.875	12.6	1.9	12.6	0.9	12.1	2.7	11.9	2.0
	22.5	−3.9	1.7	−4.9	1.9	−5.4	1.7	−2.5	2.2
Chelidonine	1.250	5.2	2.4	8.2	2.1	7.8	2.5	8.2	2.4
	15.0	−8.9	3.4	−7.1	1.6	−9.6	2.3	−6.3	2.2
Coptisine	31.25	5.2	1.9	12.3	3.5	9.5	0.8	8.0	1.9
	375	−11.7	3.2	−11.5	0.9	−10.4	3.9	−9.3	2.5
Sanguinarine	50.0	−4.8	1.7	−3.8	2.6	2.2	0.5	−4.1	1.6
	600	−6.3	1.5	−6.0	1.5	−5.3	2.3	−4.4	1.7
Chelerythrine	15.0	−13.3	3.7	−10.2	3.5	−12.7	4.2	−12.0	1.0
	180	8.2	3.6	6.5	2.9	4.5	3.1	8.4	2.0

**Table 5:** Retention time (**t_R_** ) and MS^2^ fragment ions of eight alkaloids identified in *Ch. majus.*

Compounds	Formular	t_R_ (min)	[M + H]^+^	[M]^+^	MS^2^
Magnoflorine	C_20_H_24_NO_4_	0.70	-	342	297,282,265,237,222
Protopine	C_20_H_20_NO_5_	1.63	354	-	336,305,293,275,247,188
Chelidonine	C_20_H_20_NO_5_	1.89	354	-	336,323,305,275,189
Coptisine	C_19_H_14_NO_4_	2.00	-	320	292,277,262,249
Allocryptopine	C_21_H_24_NO_5_	2.02	370	-	352,336,306,290,188
Sanguinarine	C_20_H_14_NO_4_	2.70	-	332	317,304,289,274
Berberine	C_20_H_18_NO_4_	3.07	-	336	321,320,206,292,278
Chelerythrine	C_21_H_18_NO_4_	3.93	-	348	332,318,304,290

**Table 6:** MRM ion transitions and collision energies used for the determination of five alkaloids and IS.

Compound	Precursor Ion (*m/z*)	Product Ion (*m/z*)	Collision Energy (eV)
Protopine	354.0	188.0	33
Chelidonine	354.0	275.0	28
Coptisine	320.0	261.9	36
Sanguinarine	331.9	274.0	34
Chelerythrine	348.0	304.0	32
Palmatine(IS)	352.0	308.1	30
